# Association between serum triglycerides and stroke type, severity, and prognosis. Analysis in 6558 patients

**DOI:** 10.1186/s12883-024-03572-9

**Published:** 2024-03-05

**Authors:** Naveed Akhtar, Rajvir Singh, Saadat Kamran, Sujatha Joseph, Deborah Morgan, Ryan Ty Uy, Sarah Treit, Ashfaq Shuaib

**Affiliations:** 1 Hamad Medical Corporation, Neurology, North Tower, Doha, Qatar; 2 Department of Medicine, University of Alberta, Edmonton, Alberta Canada

**Keywords:** Ischemic stroke, Triglyceride, Small vessel disease, Outcome, Major adverse cardiovascular events

## Abstract

**Background and objectives:**

Hypertriglyceridemia (HT) may increase the risk of stroke. Limited studies have shown that stroke severity and infarction size are smaller in patients with HT. We explored the relationship between triglyceride levels and stroke risk factors, severity and outcome in a large prospective database.

**Design:**

Prospective Cross-sectional study.

**Setting:**

We retrospectively interrogated the Qatar Stroke Database in all patients admitted between 2014-2022 with acute ischemic stroke and evaluated the relationship between triglyceride, diabetes, stroke severity (measured on NIHSS), stroke type (TOAST classification) and the short- (mRS at 90 days) and long-term outcomes (MACE at 1 year) in patients with HT.

**Participants:**

Six thousand five hundred fifty-eight patients ≥20 years were included in this study

**Results:**

Six thousand five hundred fifty-eight patients with ischemic stroke [mean age 54.6 ± 12. 9; male 82.1%) were included. Triglyceride levels upon admission were low-normal (≤1.1 mmol/L) in 2019 patients, high-normal (1.2-1.7 mmol/L) in 2142 patients, borderline-high (1.8-2.2 mmol/L) in 1072 patients and high (≥2.3 mmol/L) in 1325 patients. Higher triglyceride levels were associated with stroke and increased likelihood of having diabetes, obesity, active smoking, and small vessel/lacunar stroke type. An inverse relationship was noted whereby higher triglyceride levels were associated with lower stroke severity and reduced likelihood of poorer outcome (mRS 3-6) at discharge and 90 days. Long-term MACE events were less frequent in patients with higher triglyceride levels. After adjusting age, gender, diabetes, prior stroke, CAD, and obesity, multivariate analysis showed that hypertension and triglyceride levels were higher in mild ischemic strokes patients.

**Conclusions:**

Increasing triglycerides are associated with higher risk of small vessel disease and requires further prospective cohort studies for confirmation.

**Supplementary Information:**

The online version contains supplementary material available at 10.1186/s12883-024-03572-9.

## Strengths and limitations of this study

- ⇒ Effect of triglycerides on the severity of symptoms, stroke sub-types and short and long-term prognosis of patients with acute ischemic stroke with and without diabetes.
- ⇒ Cohort of patients where hypertriglyceridemia, stroke and coronary artery disease is more prevalent at younger age
- ⇒ The analytical approach can significantly reduce residual confounding.
- ⇒ The single center study setting may reduce the generalizability of study findings.
- ⇒ Retrospective analysis might have resulted in low accuracy of the comorbid profile.

## Introduction

Hypertriglyceridemia (HT) is frequently diagnosed in clinical practice, with a prevalence of ~10% worldwide [1]. The prevalence is higher in the USA (29.6%) and Europe (25.9%), which likely reflects the increase in obesity and diabetes [2]. In the Middle Eastern and South Asian population, the prevalence of HT has been estimated at 39.5% in men and 21.9% in women [3, 4]. Genetic, dietary and life-style factors contribute to the higher prevalence of HT in these populations [4]

HT is increasingly recognized as an important risk factor for both coronary artery disease (CAD) [1] and stroke [5]. Most previous studies have focused on Caucasian populations, and few have evaluated the effects of HT in the Middle-East/Asia where the incidence of stroke is higher than CAD [3]. A recent study in stroke patients from Japan showed that HT was associated with a higher incidence of intracranial atherosclerosis (ICAD) and an increase in major atherosclerotic cardiovascular events (MACE) during early follow-up [6]. South Asian and Middle Eastern populations may be particularly susceptible to the effects of HT and may manifests sequelae at a younger age [7], suggesting an urgent need for further research in this population.

Although HT contributes to the risk for stroke, several studies have reported an inverse relationship between the levels of serum triglycerides and the severity of symptoms of acute ischemic stroke [8]. This was initially reported from Glasgow in 2003 [9]. In a study of 1310 non-diabetic patients with acute stroke, increasing age, hyperglycemia and low triglycerides were associated with higher mortality. Similarly, another study with 863 ischemic stroke patients confirmed an associated between lower triglyceride levels and increased stroke severity [10]. These findings were confirmed in studies from Korea [1067 acute ischemic stroke patients] [11] and Greece [790 patients with acute ischemic stroke] [12]. Interestingly, a study of 121 patients from Zagreb showed that higher serum triglycerides was associated with a smaller volume of the ischemic stroke [13]. These are small studies that lack details on the relationship between HT and the type of stroke or long-term prognosis. There is no information on the effects of HT in the Asian or Arab population, where HT high and where stroke is more common than CAD [3].

The Qatar stroke database has prospectively collected data on all stroke admissions to the Hamad General Hospital (HGH) the main tertiary care hospital where ~95% of patients were acute stroke are admitted. The patient population is predominantly South Asian (52.8%) and Arab (32%). The database has over 15,000 patients and captures important details including risk factors, severity of symptoms, short-term fsuppland long-term prognosis [14–19]. We have previously documented high rates of dyslipidemia in this population [15, 17, 19]. For the current analysis, we had the following objectives: i) to evaluate the effects of increasing levels of serum triglycerides on the age at presentation, severity of symptoms and the type of ischemic stroke, ii) to review the relationship between HT and short and long-term prognosis following acute ischemic stroke, and iii) to determine if these relationships are independent of diabetes, which is commonly associated with HT.

## Methods

All patients admitted with a stroke, including ischemic stroke, stroke mimics, transient ischemic attack (TIA) and intracranial hemorrhage admitted to Hamad General Hospital (HGH), Doha, Qatar between January 1, 2014 through December 04, 2021 were entered into the database and the information was available for analysis. For the purpose of this study we retrospectively evaluated data on patients with a confirmed diagnosis of ischemic stroke. The details of the stroke database have previously been published [14–19]. In brief, HGH is a Joint Commission International accredited 600-bed hospital, where ~95% of all strokes in Qatar requiring hospital admission are admitted. The stroke program, also certified by the Joint Commission International, is equipped with all the necessary laboratory, neuro-radiological and neurosurgical facilities needed to manage acute stroke patients and has a 24-hour thrombolysis and thrombectomy services. All patients with a suspected diagnosis of acute stroke seen in the Emergency Department (ED) by the Stroke team to enable immediate decisions on thrombolysis and further management.

### Patient and public involvement

Patients or the public WERE NOT involved in the design, or conduct, or reporting, or dissemination plans of our research.

#### Patient characteristics and data collection

Upon identification in the ED, data was collected once confirmation of diagnosis of ischemic stroke was made using the *International Classification of Disease, 10th Edition,* definitions (I63.x). Data from emergency medical services/paramedics, immediate ED care, door-to-needle time (for thrombolysis patients), NIHSS score, length of stay (LOS), neuroimaging, post- stroke complications, in-hospital mortality, and recurrences were recorded. The modified Rankin scale (mRS) measurements were done at discharge and at 90 days [20]. The patients were classified as good (mRS of ≤0-2) or poor (mRS 3-6) outcome. We used the TOAST criteria [21] for the final diagnosis of stroke etiology. This classification defines patients in the following categories with predefined criteria; large vessel disease, small vessel or lacunar stroke (SVD), cardioembolic, and stroke of determined or undetermined etiology [20].

All blood samples for lipid levels were collected following overnight fasting which was about 12 hours in most cases. In patients presenting during the night, the fasting lipid levels were sent next day after admission. Dyslipidemia was defined as a low-density lipoprotein-cholesterol (LDL) level ≥ 3.62 mmol/L (139.98 mg/dl), high-density lipoprotein-cholesterol (HDL) level ≤ 1.03 mmol/L (39.83 mg/dl), triglycerides ≥ 1.69 mmol/L (149.69), or current treatment with a cholesterol-lowering drug [21]. In order to better understand the effects of rising triglyceride levels on ischemic stroke, we conducted our analysis in triglycerides levels of low-normal (< 1.1 mmol/l, <100 mg/dl)), high-normal (1.2-1.7 mmol/l, 101-149 mg/dl)), borderline-high (1.8-2.2 mmol/l, 150-199 mg/dl) and high (>2.3 mmol/l, > 200 mg/dl) as recommended by the National Cholesterol Educational Program [NCEP] [22]. Atrial Fibrillation (AF) was diagnosed based on electrocardiographic findings on admission or on Holter monitoring during hospitalization. Smoking was defined as current cigarette smoking. Complications monitored and recorded included aspiration pneumonia, urinary tract infection, bedsores and sepsis during hospitalization. Diabetes was diagnosed according to the American Diabetes Association (ADA) and WHO recommendations and included patients with a previous diagnosis of DM, on medication for DM or a HbA1c ≥6.5% and the diagnosis of pre-DM was based on a HbA1c of 5.7 - 6.4 % as per 2015 ADA clinical practice recommendations. Hypertension was defined as a previous systolic blood pressure ≥140 mm Hg or a diastolic blood pressure ≥ 90 mm Hg, or current treatment with antihypertensive drugs.

Diabetes is commonly seen in patients with HT. We therefore evaluated the outcomes in patients in the presence and absence of diabetes. We evaluated the relationship between triglyceride levels and stroke severity, TOAST classification for etiology, short and long-term prognosis in patients with or without diabetes.

To collect post-discharge, follow up data, the Cerner electronic medical systems were used to track patient admissions throughout the state of Qatar. We coded patient outcome using major cardiovascular events (MACE) scores based on data in the Cerner files. We collected data on recurrent stroke, post-stroke myocardial infarction (non-fatal and fatal), cardiac arrest, post-stroke cardiac revascularization and death for one year.

#### Data analysis and statistics

Descriptive results for all quantitative variables (e.g., age, systolic BP, BMI and others as shown in Table 1) were reported as mean ± standard deviation (SD). Numbers (percentage) were reported for all categorical variables (e.g., gender, diabetes, atrial fibrillation and others as shown in Table 1). The distribution of continuous variables was assessed by applying Kolmogorov Smirnov tests prior to using statistical tools.

**Table 1:** Baseline Characteristics and outcome differences of Ischemic Strokes in patients according to four categories as defined by the National Cholesterol Education Program (NCEP)

**Characteristic or Investigation**	**Total (*n*= 6558)**	**Low Normal (*n*=2019, 30.8%)**	**High Normal (*n*=2142, 32.7%)**	**Borderline High (1072, 16.3%)**	**High (*n*=1325, 20.2%)**	***P*-Value**
**Age, Mean, years**	**54.60 ± 12.890**	**56.13 ± 14.543**	**54.80 ± 12.647**	**53.59 ± 11.812**	**52.75 ± 11.006**	**<0.001**
**Male**	**5385 (82.1)**	**1583 (78.4)**	**1771 (82.7)**	**902 (84.1)**	**1129 (85.2)**	**<0.001**
**Hypertension**	**4834 (73.7)**	**1440 (71.3)**	**1596 (74.5)**	**796 (74.3)**	**1002 (75.6)**	**0.03**
**Diabetes**	**3688 (56.2)**	**933 (46.2)**	**1195 (55.8)**	**675 (63)**	**885 (66.8)**	**<0.001**
**Dyslipidemia**	**3306 (50.4)**	**780 (38.6)**	**1116 (52.1)**	**615 (57.4)**	**795 (60)**	**<0.001**
**Prior Stroke**	**754 (11.5)**	**256 (12.7)**	**241 (11.3)**	**119 (11.1)**	**138 (10.4)**	**0.20**
**Atrial Fibrillation on Admission**	**482 (7.3)**	**224 (11.1)**	**154 (7.2)**	**61 (5.7)**	**43 (3.2)**	**<0.001**
**Coronary Artery Disease**	**765 (11.7)**	**247 (12.2)**	**257 (12)**	**110 (10.3)**	**51 (11.4)**	**0.39**
**Active Smoking**	**1959 (29.9)**	**466 (23.1)**	**644 (30.1)**	**358 (33.4)**	**491 (37.1)**	**<0.001**
**Obesity (BMI ≥ 30 kg/m**^**2**^**)**	**1661 (25.3)**	**428 (21.2)**	**530 (24.7)**	**300 (28)**	**403 (30.4)**	**<0.001**
**NIHSS on admission**	**5.05 ± 5.319**	**5.84 ± 6.013**	**5.09 ± 5.252**	**4.62 ± 4.940**	**4.14 ± 4.329**	**<0.001**
**NIHSS Severity**						
**Mild (NIHHS 0-4)**	**4114 (62.7)**	**1184 (58.6)**	**1334 (62.3)**	**699 (65.2)**	**897 (67.7)**	**<0.001**
**Moderate (NIHSS 5-10)**	**1557 (23.7)**	**463 (22.9)**	**522 (24.4)**	**254 (23.7)**	**318 (24.0)**	
**Severe (NIHSS >10)**	**887 (13.5)**	**372 (18.4)**	**286 (13.4)**	**119 (11.1)**	**110 (8.3)**	
**RBS on admission**	**9.53 ± 4.91**	**8.52 ± 4.20**	**9.36 ± 4.71**	**10.23 ± 5.41**	**10.79 ± 5.43**	**<0.001**
**HbA1c %**	**7.47 ± 2.39**	**6.81 ± 2.06**	**7.36 ± 2.25**	**7.89 ± 2.56**	**8.30 ± 2.59**	**<0.001**
**Serum Cholesterol**	**4.86 ± 1.59**	**4.33 ± 1.47**	**4.85 ± 1.79**	**5.08 ± 1.34**	**5.49 ± 1.31**	**<0.001**
**Serum HDL**	**1.01 ± 0.29**	**1.13 ± 0.33**	**1.01 ± 0.26**	**0.95 ± 0.25**	**0.89 ± 0.23**	**<0.001**
**Serum LDL**	**3.05 ± 1.13**	**2.78 ± 1.03**	**3.15 ± 1.11**	**3.22 ± 1.21**	**3.16 ± 1.17**	**<0.001**
**Systolic Blood Pressure**	**157.70 ± 30.23**	**154.47 ± 30.48**	**158.50 ± 29.69**	**159.19 ± 30.45**	**160.12 ± 30.14**	**<0.001**
**Diastolic Blood Pressure**	**91.19 ± 19.25**	**89.31 ± 18.77**	**91.32 ± 19.47**	**92.48 ± 20.21**	**92.81 ± 18.59**	**<0.001**
**Length of Stay**	**5.41 ± 6.14**	**6.10 ± 6.89**	**5.29 ± 5.58**	**5.16 ± 5.99**	**4.75 ± 5.78**	**<0.001**
**Complications during admission**	**422(6.4)**	**185(9.2)**	**131(6.1)**	**52(4.9)**	**54(4.1)**	**<0.001**
**Aspiration Pneumonia**	**199(3)**	**87(4.3)**	**64(3)**	**23(2.1)**	**25(1.9)**	**<0.001**
**Urosepsis**	**146(2.2)**	**66(3.3)**	**42(2)**	**22(2.1)**	**16(1.2)**	**<0.001**
**Bed sores**	**24(0.4)**	**13(0.6)**	**5(0.2)**	**2(0.2)**	**4(0.3)**	**0.09**
**Sepsis**	**136(2.1)**	**58(2.9)**	**42(2)**	**15(1.4)**	**21(1.6)**	**0.02**
**Deep venous thrombosis**	**5(0.1)**	**3(0.1)**	**0**	**2(0.2)**	**0**	**0.13**
**Symptomatic bleed**	**11(0.2)**	**4(0.2)**	**5(0.2)**	**2(0.2)**	**0**	**0.41**
**Prognosis –Discharge**						
**Good (mRS 0-2)**	**3959 (60.4)**	**1118 (55.4)**	**1296 (60.5)**	**680 (63.4)**	**865 (65.3)**	**<0.001**
**Poor (mRS 3-6)**	**2599 (39.6)**	**901 (44.6)**	**846 (39.5)**	**392 (36.6)**	**460 (34.7)**	
**Prognosis 90-Days(*n*= 5021,76.6%)**						
**Good (mRS 0-2)**	**3543 (70.6)**	**1006 (65)**	**1164 (71.5)**	**613 (73.9)**	**760 (74.9)**	**<0.001**
**Poor (mRS 3-6)**	**1478 (29.4)**	**542 (35.0)**	**465 (28.5)**	**216 (26.1)**	**255 (25.1)**	
**Mortality – At Discharge**	**70 (1.1)**	**29(1.4)**	**32(1.5)**	**4 (0.4)**	**5 (0.4)**	**<0.001**
**Mortality at 90-Days**	**179 (3.6)**	**79 (5.1)**	**64 (3.9)**	**13 (1.6)**	**23 (2.3)**	**<0.001**
**TOAST Classification**						
**Small Vessel Disease**	**3182 (48.5)**	**883 (43.7)**	**1018 (47.5)**	**537 (50.1)**	**744 (56.2)**	**<0.001**
**Large Vessel Disease**	**1385 (21.1)**	**426 (21.1)**	**461 (21.5)**	**241 (22.5)**	**257 (19.4)**	
**Cardioembolic**	**1207 (18.4)**	**436 (21.6)**	**414 (19.3)**	**173 (16.1)**	**184 (13.9)**	
**Stroke of Determined Origin**	**489 (7.5)**	**174 (8.6)**	**161 (7.5)**	**69 (6.4)**	**85 (6.4)**	
**Stroke of Undetermined Origin**	**295 (4.5)**	**100 (5)**	**88 (4.1)**	**52 (4.9)**	**55 (4.2)**	
**One Year Outcome**						
**Recurrent Stroke (Ischemic or Hemorrhagic)**	**122 (2.4)**	**37 (2.4)**	**38 (2.3)**	**20 (2.4)**	**27 (2.5)**	**0.98**
**Post-Stroke MI (fatal or non-fatal)**	**39 (0.8)**	**4 (0.3)**	**16 (1)**	**10 (1.2)**	**9 (0.8)**	**0.04**
**All-Cause Mortality**	**228 (4.5)**	**103 (6.7)**	**73 (4.4)**	**27 (3.2)**	**25 (2.4)**	**<0.001**
**Post-Stroke Cardiac Arrest**	**55 (1.1)**	**25 (1.6)**	**13 (0.8)**	**7 (0.8)**	**10 (0.9)**	**0.09**
**Post-Stroke Cardiac Revascularization (CABG or PCI)**	**39 (0.8)**	**10 (0.70)**	**12 (0.7)**	**7 (0.8)**	**1 0(0.9)**	**0.85**
**Total MACE at one year**	**401 (6.1)**	**152 (7.5)**	**126 (5.9)**	**57 (5.3)**	**66 (5)**	**0.01**

Triglyceride values – Low normal – 1.1 mmol/L or less, High normal- 1.2-1.7 mmol/L, Borderline High- 1.8-2.2 mmol/L, and High levels- 2.3 mmol/L or more. IV- intravenous, *NIHSS* National Institute of Health Stroke Scale, *RBS* Random blood sugar, *HDL* High density lipoprotein, *LDL* Low density lipoprotein, *BMI* Body Mass Index, *mRS* Modified Rankin Score, *CABG* Coronary artery bypass Graft, *PCI* Percutaneous Coronary Intervention, *MACE* Major Cardiac Adverse Event

One Way ANOVA was applied to test for significant differences across triglyceride levels for all continuous variables whereas; chi-square tests were used for all categorical variables. Independent sample t-test was used to compare the average for all the quantitative variables between patients with and without diabetes and Pearson Chi-Square test or Fisher Exact test was used to compare the proportion of all qualitative variables between patients with and without diabetes. All covariates were selected based significance on univariate analysis.

A multiple logistic regression analysis for the variables significant at univariate analysis was performed to determine if demographic (e.g., age, gender etc.) and clinical characteristics were associated with those who had mild stroke (NIHSS **≤**=4) in comparison to moderate -severe strokes (Table 2). The Wald test was computed on each predictor to determine which were significant. Adjusted Odds ratio and 95% confidence interval for the odds ratio were reported (Table 3).

**Table 2:** Baseline Characteristics and outcome of ischemic stroke patients with minor strokes (NIHSS ≤4) on admission

**Characteristic or Investigation**	**Total (*n*= 6658)**	**Mild Stroke (*n*= 4114, 62.7 %)**	**Moderate to Severe Stroke (*n*= 2444, 37.3%)**	***P*-Value**
**Age, Mean, years**	**54.4 ±12.8**	**54.4 ±12.3**	**54.9 ±13.7**	**<0.001**
**Male Sex**	**5385 (82.1)**	**3401 (82.7)**	**1984 (81.2)**	**0.13**
**Diabetes**	**3688 (56.2)**	**2306 (56.1)**	**1382 (56.5)**	**0.69**
**Hypertension**	**4834 (73.7)**	**3102 (75.4)**	**1732 (70.9)**	**<0.001**
**Dyslipidemia**	**3306 (50.4)**	**2150 (52.3)**	**1156 (47.3)**	**<0.001**
**Prior Stroke**	**754 (11.5)**	**461 (11.2)**	**293 (12.0)**	**0.34**
**Coronary Artery Disease**	**765 (11.7)**	**454 (11.0)**	**311 (12.7)**	**0.04**
**Atrial Fibrillation**	**482 (7.3)**	**225 (5.5)**	**257 (10.5)**	**<0.001**
**Active Smoking**	**1959 (29.9)**	**1225 (29.8)**	**734 (30.0)**	**0.83**
**Obesity (BMI ≥ 30 kg/m**^**2**^**)**	**1661 (25.3)**	**1063 (25.8)**	**598 (24.5)**	**0.22**
**Hypertriglyceridemia**				
**Low Normal (≤ 1.1mmol/L)**	**2019 (30.8)**	**1184 (28.8)**	**835 (34.2)**	**<0.001**
**High Normal (1.2 – 1.7 mmol/L)**	**2142 (32.7)**	**1334 (32.4)**	**808 (33.1)**	
**Borderline High (1.8 – 2.2 mmol/L)**	**1072 (16.3)**	**699 (17.0)**	**373 (15.3)**	
**High (≥ 2.3 mmol/L)**	**1325 (20.2)**	**897 (21.8)**	**428 (17.5)**	
**IV Thrombolysis given**	**779 (11.9)**	**123 (3.0)**	**656 (26.8)**	**<0.001**
**Complications during admission**	**422 (6.4)**	**97 (2.4)**	**325 (13.3)**	**<0.001**
**Prognosis 90-Days (n= 5021)**				
**Good (mRS 0-2)**	**3543 (70.6)**	**2692 (84.1)**	**851 (46.7)**	**<0.001**
**Poor (mRS 3-6)**	**1478 (29.4)**	**508 (15.9)**	**970 (53.3)**	
**Mortality at 90-Days**	**179 (3.6)**	**41 (1.3)**	**138 (7.6)**	**<0.001**
**One Year Outcome**				
**Recurrent Stroke (Ischemic or Hemorrhagic)**	**122 (2.4)**	**84 (2.6)**	**38 (2.1)**	**0.28**
**Myocardial Infarction (fatal or non-fatal)**	**39 (0.8)**	**27 (0.8)**	**12 (0.7)**	**0.51**
**All-Cause Mortality**	**228 (4.5)**	**61 (1.9)**	**167 (9.1)**	**<0.001**
**Cardiac Arrest**	**55 (1.1)**	**17 (0.5)**	**38 (2.1)**	**<0.001**
**Cardiac Revascularization (CABG or PCI)**	**39 (0.8)**	**26 (0.8)**	**13 (0.7)**	**0.76**
**Total MACE at one year**	**401 (6.1)**	**180 (4.4)**	**221 (9.0)**	**<0.001**

Mild Stroke (NIHSS 4 or less), Moderate-Severe stroke (NIHSS 5 or more) on admission

**Table 3:** Multivariate Logistic regression analysis showing factors associated to minor ischemic strokes (NIHSS ≤ 4)

**Variable**	**Adjusted Odds Ratio**	**95.0% CI**		***P* -value**
		**Lower**	**Upper**	
**Hypertension**	**1.33**	**1.13**	**1.56**	**<0.001**
**Atrial Fibrillation**	**0.47**	**0.37**	**0.59**	**<0.001**
**TOAST Classification**				
**Small Vessel Disease**	**1.0**	**-**	**-**	**<0.001**
**Large Vessel Disease**	**0.12**	**0.10**	**0.16**	**<0.001**
**Cardio Embolic**	**0.15**	**0.12**	**0.19**	**<0.001**
**Stroke of Determined Origin**	**0.16**	**0.12**	**0.21**	**<0.001**
**Stroke of Undetermined origin**	**0.31**	**0.21**	**0.47**	**<0.001**
**Hypertriglyceridemia**	**1.26**	**1.15**	**1.38**	**<0.001**

Results are adjusted to age (years), gender, diabetes, prior stroke history, coronary artery disease, active smoker, and obesity

A p-value 0.05 (two tailed) was considered statistically significant level. SPSS 21·0 statistical package was used for the analysis.

## Results

During the time period of analysis (January 2014-December 2021), there were 14,768 patients entered into the acute stroke database. After exclusion of patients with ICH (1549), TIAs (1413), stroke mimics (4302), and cerebral venous thrombosis (204), there were 7300 patients with a diagnosis of ischemic stroke. After excluding 742 patients in whom the data was not complete, we were left with 6558 patients for the study.

### Patient characteristics

**Fig. 1:**
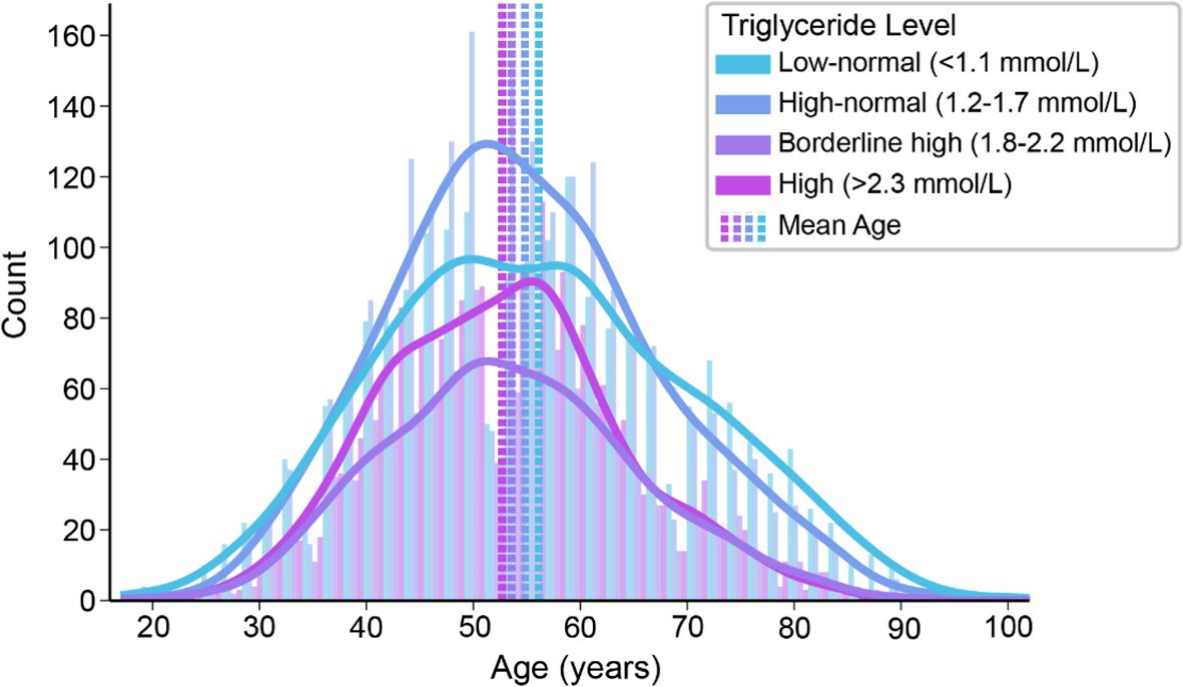
Histograms and corresponding kernel density estimates of the age distribution of subjects based on triglyceride level (low-normal <1.1 mmol/L; borderline high 1.2-1.7 mmol/L; high-normal 1.8-2.2 mmol/L or high >2.3 mmol/L)

There was a predominance of men in this sample (82.1%), reflecting the high percentage of male expatriate workers in Qatar (http://www.mdps.gov.qa/english/population). The mean age of the 6558 patients at presentation was 54.6 ± 12.9. When divided into four triglyceride groups as per NCEP recommendations, there were 2019 [30.8%] patients in the low normal group, 2142 [32.7%] patients in the high normal, 1072 [16.3%] in the borderline high and 1325 [20.2%] patients in the high groups. ANOVA indicated an effect of triglyceride level on age at presentation, whereby higher triglyceride levels were associated with younger age (low normal: 56.1 ± 14.5, high normal: 54.8 ± 12.6 borderline high: 53.6 ± 11.8, high: 52.8 ± 11.0, *p*<0.001) as shown in Fig. 1.

Patients with HT were significantly more likely to have diabetes, obesity (BMI ≥ 30 kg/m^2^), be active smokers, and have abnormalities in other lipids, but were not more likely to have hypertension, previous stroke or CAD as shown in Table 1.

### Stroke severity at admission

**Fig. 2:**
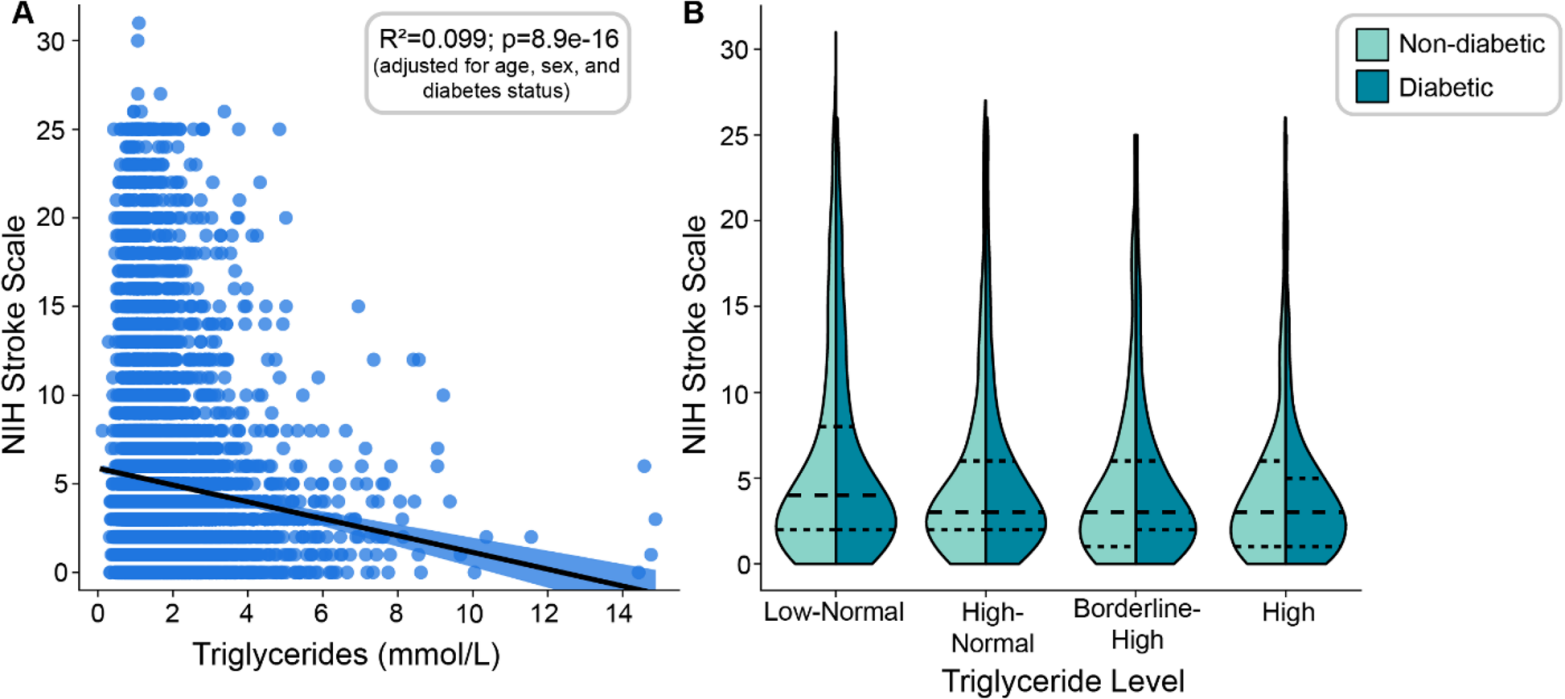
**A** Linear regression indicating a significant negative relationship between NIH stroke scale and serum triglycerides (lower NIHSS associated with higher triglycerides) in all subjects (*n*=6558), controlling for age, sex and diabetes status. **B** Split violin plot showing the median, interquartile range and spread of NIH stroke scales when subjects are divided into groups based on triglyceride level (x axis) and diabetes status (left and right half of each violin)

The severity of stroke symptoms, as measured with the NIHSS, was negatively correlated with serum triglyceride values (Fig. 2A), even after covarying for diabetes status. This relationship was also evident when NIHSS was compared across categories of triglyceride level (Fig. 2B). Most patients had mild symptoms (percentage of patients with ischemic stroke presenting with an NIHSS of less **≤** 5: 4114 [62.7%]). This is typical of the severity of stroke in the local population where a high proportion of subjects have untreated hypertension and diabetes [15–19].

Medical complications, especially aspiration and urosepsis differed across triglyceride groups (higher likelihood with lower triglycerides) and the length of stay (LOS) in hospital was also longer in these patients, once again a reflection of more severe stroke as shown in Tables 1 and 2.

**Fig. 3:**
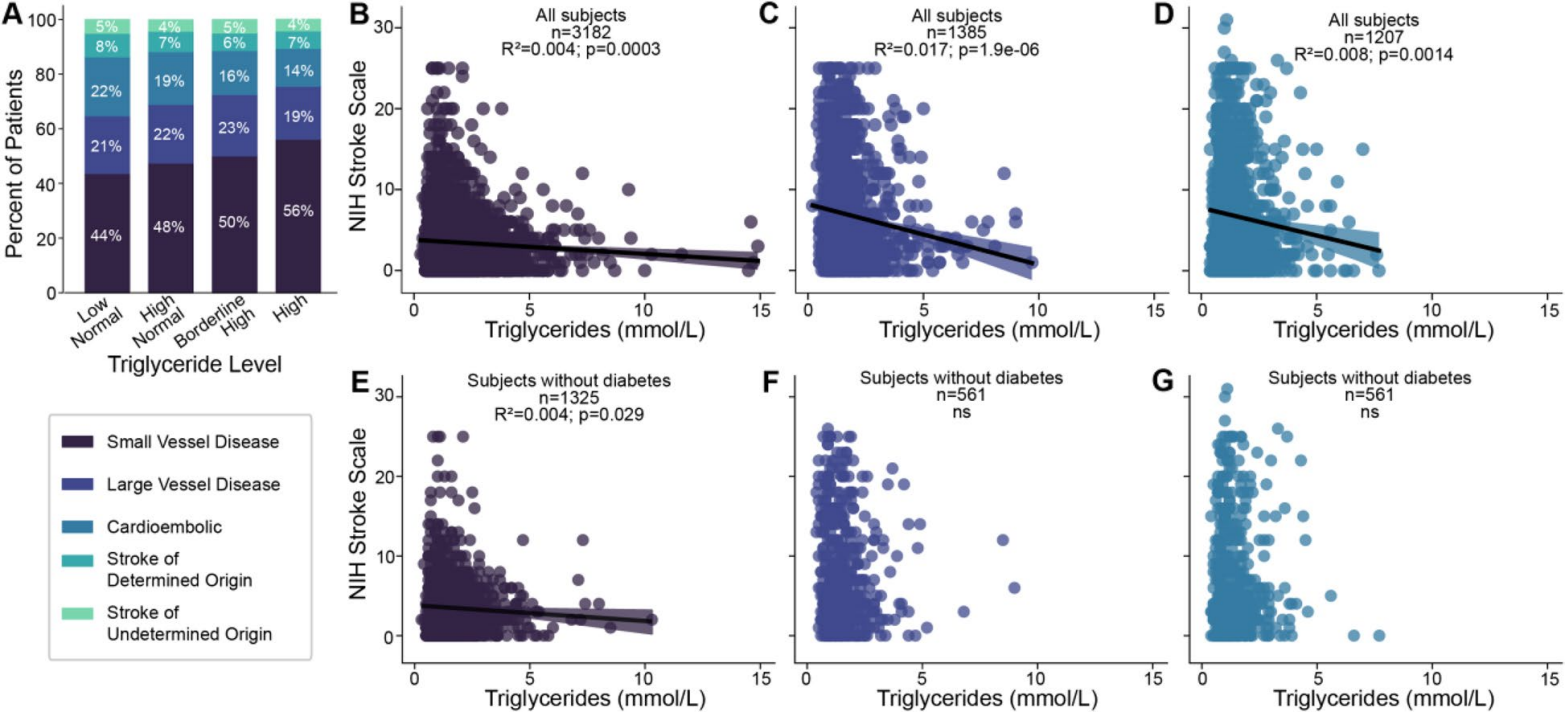
Scatterplots and linear regression dividing the sample into groups based on diabetes status (top row: non-diabetics, bottom row: diabetics) and stroke type based on the TOAST classification system

We used the TOAST classification to define the final diagnosis of the patients (Table 3). As seen in Fig. 3, the percentage of patients with SVD/lacunar strokes increased with rising levels of triglycerides. In addition, NIHSS scores decreased with increasing serum triglycerides in patients with small vessel disease/lacunar stroke, large vessel disease and cardioembolic stroke (Fig. 3). The higher proportion of patients with lacunar stroke likely explains the milder severity of neurological symptoms at presentation, fewer complications and shorter LOS in patients with HT.

In order to determine the relationship between HT and SVD/lacunar stroke, we did additional analysis, of an increase in triglycerides on the severity of symptoms and outcome in patients with SVD/lacunar stroke. There were 3182 patients with SVD/lacunar strokes available for analysis. Patients presenting with SVD/lacunar stroke had similar characteristics to the larger cohort with increasing serum triglyceride values. Increasing triglyceride levels were associated with milder stroke symptoms (as measured on NIHSS) at presentation and fewer complications while in hospital and better prognosis as shown in Supplemental Table 1.

### Hypertriglyceridemia in the absence of diabetes

As diabetes is a well-recognized risk factor for SVD/lacunar, we did additional analyses of the data in patients without diabetes. After excluding 3688 patients with diabetes, the analysis was repeated in 2870 remaining patients. Non-diabetic patients had similar characteristics to the entire cohort. Increasing levels of triglycerides was associated with milder symptoms at presentation (NIHSS 0-4 in 61.5 vs. 66.7, p<0.002). Patients without diabetes with increasing triglyceride levels were also more likely to have SVD/lacunar stroke as the etiology, shorter LOS and fewer medical complications and better prognosis at discharge and 90-days. Further details are shown in Supplemental Table 2.

### Disability at discharge and 90-days

**Fig. 4:**
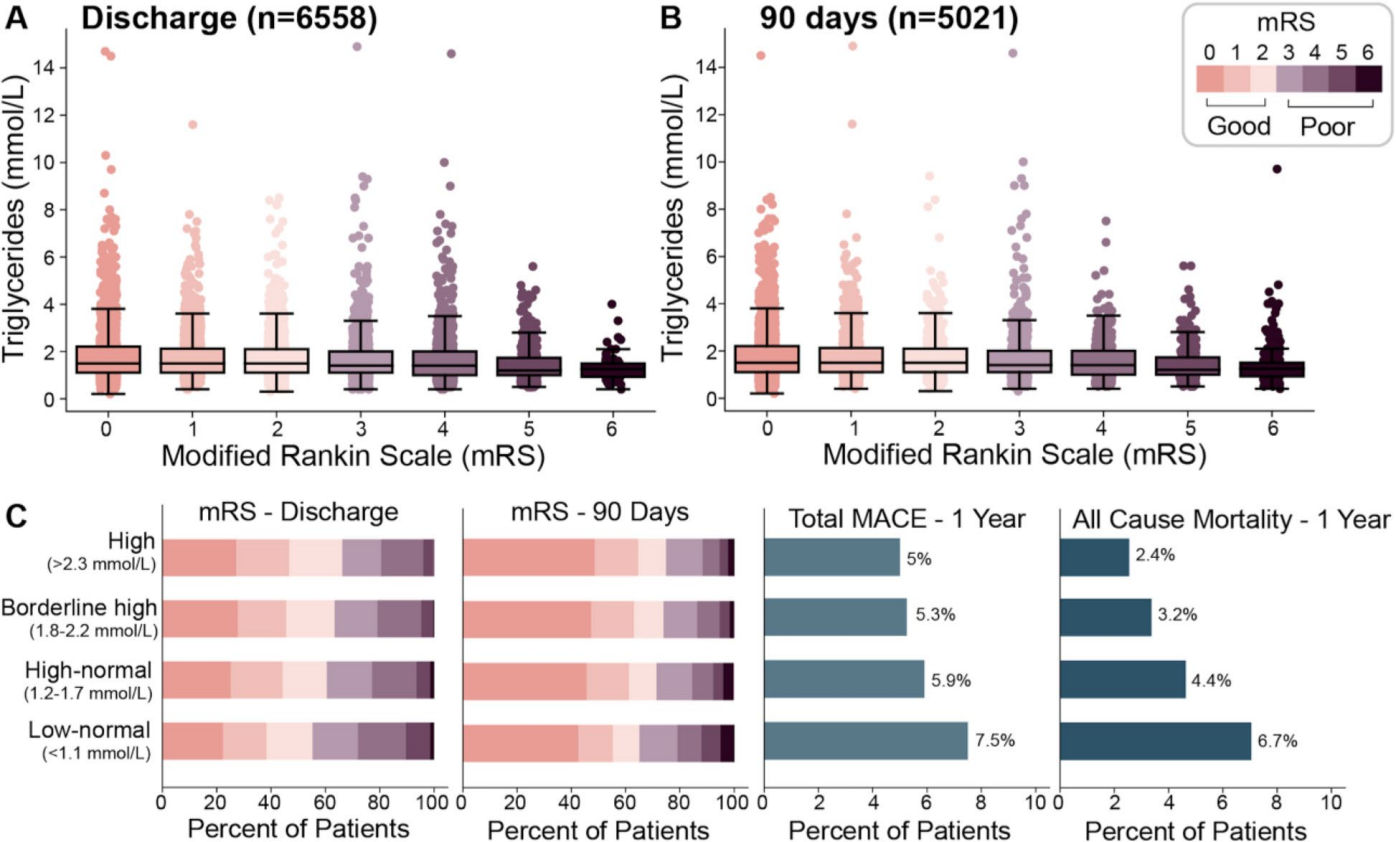
Combined strip and box-plots of modified Rankin scales (mRS) versus triglyceride levels at discharge (**A**) and 90 days post stroke (**B**), as well as stacked bar charts indicating the proportion of patients with each mRS score (0-6) by triglyceride group at discharge and 90 days post stroke (**C**). At both discharge and 90 days the participants with mRS scores of 5 or 6 (indicating the poorest outcome) had the lowest triglyceride levels. Likewise, highest proportion of subjects with mRS scores indicating a poor outcome (3-6) were in the low-normal triglyceride group

Disability at discharge and at 90 days was measured on the mRS (mRS of 3-6 were considered as poor outcome). The percentage of patients with mRS of 3-6 at discharge and 90-days decreased with increasing triglyceride levels as shown in Table 1 and Fig. 4. The difference in the 90 days poor outcome remained significant when the analysis was done in patients with stroke and no diabetes. Mortality in hospital and at 90 days was also highest in the groups with lower triglyceride levels Table 1; Fig. 4.

### Risk of recurrent stroke and MACE

The overall rate of recurrent stroke during the year following the stroke was 3.1%. Rates of recurrent strokes did not differ across triglyceride groups. Similarly, there was no differences in the risk of post-stroke myocardial infarction or cardiac revascularization between the four groups. All-cause mortality was however higher in patients with lower triglycerides (6.7% for normal-low, 4.4% for high-normal, 3.2% for borderline high and 2.4% for patients with high triglycerides). MACE was also significantly higher in the patients with lowest triglycerides. The details are shown in Table 1. These differences in mortality and MACE were not apparent once patients with diabetes were excluded from the analysis, as shown in Supplemental Table 2.

#### Multivariate analysis

Significant variables from the univariate analysis, including age at the time of presentation, gender, triglyceride levels, diabetes, hypertension, prior stoke history, obesity, CAD and active smoking and TOAST classification were used in multivariate logistic regression analysis to assess the associated factors to mild stroke (NIHSS) in comparison to moderate-severe stroke at the time of admission, presented in Table 3 and Supplemental Table.

Variables including age, gender, diabetes, prior stroke history, coronary artery disease, active smoking obesity were adjusted in the multivariate analysis. Hypertension (adjusted odds ratio (aOR) 1.17 [ 95% CI 1.03 – 1.32] *p*= 0.01) was found more in mild stroke patients in comparison to moderate-severe stroke. Also, milder stroke severity was found with borderline (aOR 1.24 [ 95% CI 1.08 – 1.45] *p*= 0.01) and high levels (aOR 1.29 [95% CI 1.12 – 1.51] *p*= 0.001) in comparison to low normal HT levels. Similarly, higher levels of triglycerides were associated with lower frequency of large vessel disease (aOR 0.33 [95% CI 0.29 – 0.38], *p*=0.001), cardio embolic (aOR 0.42 [95% CI 0.36 – 0.49], *p*=0.001), stroke of determined origin (aOR 0.38 [95% 0.31 – 0.46], *p*=0.001) and stroke of undetermined origin (aOR 0.57 [95% CI 0.45 – 0.74], *p*=0.001) in comparison to small vessel disease.

Another multivariate logistic regression analysis where hypertriglyceridemia was taken as continuous variable for small vessel disease etiology demonstrated that hypertriglyceridemia was interdentally associated with small vessel disease after adjusting independent variables such as diabetes, dyslipidemia, obesity and prior stroke as well as age, sex, HTN, NIHSS on admission, coronary artery disease, and atrial fibrillation (Supplementary Table).

## Discussion

This study evaluates the effects of increasing levels of triglycerides on the severity of symptoms, stroke sub-types and the short and long-term prognosis. As diabetes is frequently seen in patients with high triglyceride values, we did additional analysis in patients without diabetes. An important feature of our study is the evaluation of the detrimental effects of HT in patients from the Middle Eastern and South Asia. In these populations, HT is very common and stroke and CAD presents at an earlier age [3, 4, 15, 23–26].

In our study, there was correlation of higher triglyceride levels with having acute strokes. This was evident in patients with or without diabetes. Similar to previous observation [9–13, 27–30], we also noted that the severity of acute stroke in patients with HT was milder. We believe that this can be best explained by a higher incidence of acute SVD/lacunar strokes in the HT population. Previous studies, while concentrating on the severity of neurological symptoms, have not documented the ischemic stroke sub-types. The better outcome at 90-days follow-up in HT is also likely related to the higher rates of SVD/lacunar stroke. During a follow-up of one year, MACE events were significantly were higher in HT patients, suggesting a higher atherosclerosis risk.

Diabetes is a very common risk factor for stroke in the Middle Eastern and South East Asian population [4, 5, 24–26, 31] and was seen in 56% of our patients and likely contributed to the younger age at onset of stroke. As diabetes is frequently associated with SVD, we also evaluated the relationship between triglyceride levels and stroke in patients with no diabetes. Similar to the total cohort, patients without diabetes also had strokes at a younger age, had milder symptoms and a significantly higher incidence of SVD/lacunar strokes. Whereas the 90- days outcome was better in patients with HT, the one-year MACE did not show any significant differences in patients with increasing triglyceride values.

Hypertriglyceridemia most commonly results from genetic factors or from consumption of food high fructose or fatty products. The higher prevalence in USA and Europe likely reflects the higher rates of obesity and diabetes [2]. Hypertriglyceridemia is seen more frequently in the Arab and South Asian population [4]. Triglycerides remain high in the South Asian individuals after they immigrate to UK and contribute to the higher risk of coronary artery disease [7]. Genetic and dietary factors account for most of the HT in this population. The effects of HT have been studied mostly in coronary artery disease and there is convincing evidence that rising levels of triglycerides leading to higher incidence of coronary and systemic atherosclerosis [32, 33].

The relationship between stroke and HT was studied in the Copenhagen City Heart Study. The study followed 13,956 city dwellers between 1976 and 2007. Stroke developed in 1529 individuals. The risk of stroke increased with increasing levels of triglyceride levels [34]. Similar trends have been noted from Asia-Pacific region, India, China, American Aboriginal population and Europe [1]. The increased risk for stroke remained after adjustment for age, body mass index, family history, systolic blood pressure, diabetes and obesity [34, 35].

Does HT contribute to an increase in small vessel disease? There is evidence that HT may increase asymptomatic small vessel disease in the population. Two observation studies from France reported on the cross-sectional association of lipid abnormalities and asymptomatic cerebral small vessel disease as measured on MRI. All subjects were free of coronary artery or cerebrovascular disease at the time of inclusion into the study. The 3C-Dijon study comprised of 1842 subjects and the Epidemiology of Vascular Aging Study compromised of 766 subjects. Increasing serum triglycerides was associated with an increasing burden of SVD. This was not evident with abnormalities in other lipids. The association of SVD and HT was attenuated but maintained after adjusting for other vascular risk factors or inflammatory markers [36]. The underlying mechanisms for the increase in asymptomatic SVD with HT are not fully understood. Triglycerides have pro-inflammatory properties [37]. These researchers speculated that the SVD may be related to triglyceride-induced chronic inflammation. An increase in triglycerides is associated with blood-brain dysfunction, which may contribute to the development of SVD [38]. Importantly, HT has also been shown to affect compliance of cerebral small vessels and may result in chronic white matter hypo-perfusion, which may lead to SVD [36, 39]. Given the increased prevalence of HT, especially in the South Asian and Middle Eastern population, the increase in the development of SVD requires further study. Treatment of HT with omaga-3 agent icosapent ethyl in a large phase-III trial was shown to significantly reduce the risk of CAD and stroke in actively treated patients and may be considered in patients with HT to reduce asymptomatic SVD, and the risk of recurrent stroke and heart disease [40].

Weir et al first reported the association between serum triglyceride levels and acute stroke in 2003 [9]. Paradoxically, lower levels of triglycerides were associated with poor outcome in acute stroke. The study involved 1310 non-diabetic acute stroke patients. Age, hyperglycemia and lower triglyceride levels independently predicted higher mortality [9]. A number of subsequent reports from across the globe have confirmed similar observations between serum triglycerides and stroke prognosis in patients with and without diabetes [10–13, 27–30]. In addition, imaging studies have shown that patients with HT have smaller size of ischemic infarction [13]. The better prognosis with HT in older patients reported from China was also likely related to higher rates of SVD [27]. In patients with HT, diabetes further increases small vessel disease and lacunar stroke [30]. Our study with 6558 ischemic stroke patients provides a comprehensive understanding of the relationship between acute stroke, HT and diabetes. The incidence of diabetes and HT was high in this population and the database allows for important conclusions that were not possible with previous studies. Our study shows that together with diabetes and hypertension, HT is an important contributor of acute SVD/lacunar stroke and required aggressive treatment to prevent recurrences.

There are limitations in our study. As this is a single center data, the design may limit the generalizability of findings. The study population is distinctive comprising mainly of Arab and Asian community, which limits generalizability of the results. Also, the retrospective analysis introduces the possibility of imprecise comorbidity data. Although the lipid levels were on fasting specimens following the acute stroke, these are a single-time measurement and may not accurately reflect HT. It is important to note that our study population is a predominantly young male expatriate and may not reflect cerebrovascular disease the general population. Finally, the association of HT to stroke outcome is limited predominantly to Arab and South East Asia populations. The effects cannot be genialized to other populations. The MACE follow-up was for one year, which may not be enough to evaluate the damaging effects of HT in this high-risk population.

In conclusion, we can make the following important observations from our study with more than 6550 patients. HT contributes to an increase in the percentage of patients with SVD/lacunar strokes. This is evident in patients with and without diabetes. The higher rates of SVD/lacunar strokes are most likely responsible for the milder symptoms and lower rates of medical complications in patients with HT and acute stroke. This is also the most likely reason for shorter hospital stay and higher rates of patients with higher rates of 90-days mRS of 0-2 in the HT group. Our study and the previous reports that HT is an important and perhaps under-recognized contributor to acute stroke. The increased SVD/lacunar stroke requires strategies that are targeted to this specific population [41].

### Supplementary Information

**Supplementary Material 1.** 

## Data Availability

All data generated or analysed during this study are included in this published article and its supplementary information files.
