# Supplementary material for: Association between serum triglycerides and stroke type, severity, and prognosis. Analysis in 6558 patients
Source: BMC Neurol. 2024 Mar 5;24:88. doi: 10.1186/s12883-024-03572-9 (PMC10913234; doi:10.1186/s12883-024-03572-9)
Supplement: Supplementary file 1 — Supplementary Material 1. [file 12883_2024_3572_MOESM1_ESM.pdf]

Supplemental table 1- Baseline Characteristics and outcome of ischemic stroke patients admitted with small vessel disease etiology

Characteristic or Investigation	Total (n= 3182)	Low Normal (n=883, 27.7%)	High Normal (n=1018, 32.0%)	Borderline High (537, 16.9%)	High (n=744, 23.4%)	P-Value
Age, Mean, years	55.4 ±12.1	57.2 ±13.4	55.7 ±11.9	53.5 ±10.9	55.4 ±12.1	<0.001
Male Sex	2643 (83.1)	711 (80.5)	845 (83.0)	451 (84.0)	636 (85.5)	0.06
Diabetes	1857 (58.4)	433 (49.0)	576 (56.6)	346 (64.4)	502 (67.5)	<0.001
Hypertension	2541 (79.9)	681 (77.1)	831 (81.6)	436 (81.2)	593 (79.7)	0.08
Prior Stroke	373 (11.7)	117 (13.3)	110 (10.8)	64 (11.9)	82 (11.0)	0.36
Atrial Fibrillation	109 (3.4)	47 (5.3)	34 (3.3)	16 (3.0)	12 (1.6)	<0.001
Coronary Artery Disease	297 (9.3)	88 (10.0)	87 (8.5)	50 (9.3)	72 (9.7)	0.74
Active Smoking	966 (30.4)	212 (24.0)	309 (30.4)	170 (31.7)	275 (37.0)	<0.001
Obesity (BMI ≥ 30 kg/m^2)	802 (25.2)	173 (19.6)	257 (25.2)	160 (29.8)	212 (28.5)	<0.001
NIHSS on admission	3.5 ±3.3	3.8 ±3.8	3.5 ±3.2	3.4 ±3.1	3.1 ±2.7	<0.001
NIHSS Severity						
Mild (NIHHS 0-4)	2370 (74.5)	651 (73.7)	758 (74.5)	398 (74.1)	563 (75.7)	0.002
Moderate (NIHSS 5-10)	693 (21.8)	180 (20.4)	222 (21.8)	124 (23.1)	167 (22.4)	
Severe (NIHSS >10)	119 (3.7)	52 (5.9)	38 (3.7)	15 (2.8)	14 (1.9)	
IV Thrombolysis given	208 (6.5)	46 (5.2)	63 (6.2)	45 (8.4)	54 (7.3)	0.09
Complications during admission	73 (2.3)	34 (3.9)	23 (2.3)	9 (1.7)	7 (0.9)	<0.001
Prognosis 90-Days (n= 2458)						
Good (mRS 0-2)	1920 (78.1)	501 (73.1)	620 (79.3)	336 (81.2)	463 (80.2)	0.003
Poor (mRS 3-6)	538 (21.9)	184 (26.9)	162 (20.7)	78 (18.8)	114 (19.8)	
Mortality at 90-Days	40 (1.6)	22 (3.2)	11 (1.4)	2 (0.5)	5 (0.9)	<0.001
One Year Outcome						
Recurrent Stroke (Ischemic or Hemorrhagic)	60 (2.4)	14 (2.0)	18 (2.3)	10 (2.5)	18 (3.0)	0.70
Myocardial Infarction (fatal or non-fatal)	14 (0.6)	3 (0.4)	6 (0.8)	2 (0.5)	3 (0.5)	0.84
All-Cause Mortality	56 (2.3)	27 (3.9)	16 (2.0)	7 (1.7)	6 (1.0)	0.003
Cardiac Arrest	10 (0.4)	5 (0.7)	2 (0.3)	2 (0.5)	1 (0.2)	0.37
Cardiac Revascularization (CABG or PCI)	15 (0.6)	6 (0.9)	6 (0.8)	1 (0.2)	2 (0.3)	0.43
Total MACE at one year	135 (4.2)	48 (5.4)	41 (4.0)	19 (3.5)	27 (3.6)	0.21

Triglyceride values – Low normal – 1.1 mmol/L or less, High normal- 1.2-1.7 mmol/L, Borderline High- 1.8-2.2 mmol/L, and High levels- 2.3 mmol/L or more.
IV- intravenous, NIHSS- National Institute of Health Stroke Scale, RBS – Random blood sugar, HDL- High density lipoprotein, LDL Low density lipoprotein, BMI- Body Mass Index, mRS- Modified Rankin Score, CABG- Coronary artery bypass Graft, PCI- Percutaneous Coronary Intervention, MACE- Major Cardiac Adverse Event

Supplemental table 2- Baseline Characteristics and outcome of non-Diabetic ischemic stroke patients.

Characteristic or Investigation	Total (n= 2870)	Low Normal (n=1086, 37.8%)	High Normal (n=947, 33.0%)	Borderline High (397, 13.8%)	High (n=440, 15.3%)	P-Value
Age, Mean, years	50.5 ±13.1	52.2 ±14.9	50.2 ±12.3	48.9 ±11.6	48.1 ±10.4	<0.001
Male Sex	2436 (84.9)	860 (79.2)	814 (86.0)	354 (89.2)	408 (92.7)	<0.001
Hypertension	1834 (63.9)	666 (61.3)	631 (66.6)	253 (63.7)	284 (64.5)	0.09
Prior Stroke	191 (6.7)	94 (8.7)	60 (6.3)	18 (4.5)	19 (4.3)	0.003
Atrial Fibrillation	203 (7.1)	107 (9.9)	64 (6.8)	20 (5.0)	12 (2.7)	<0.001
Coronary Artery Disease	183 (6.4)	79 (7.3)	53 (5.6)	26 (6.5)	25 (5.7)	0.42
Active Smoking	889 (31.0)	253 (23.3)	311 (32.8)	143 (36.0)	182 (41.4)	<0.001
Obesity (BMI ≥ 30 kg/m^2)	614 (21.4)	204 (18.8)	206 (21.8)	92 (23.8)	112 (25.5)	0.02
NIHSS on admission	5.2 ±5.5	5.8 ±6.1	5.0 ±5.3	4.6 ±4.9	4.4 ±4.8	<0.001
NIHSS Severity						
Mild (NIHHS 0-4)	1808 (63.0)	646 (59.5)	604 (63.8)	264 (66.5)	294 (66.8)	0.002
Moderate (NIHSS 5-10)	643 (22.4)	243 (22.4)	214 (22.6)	89 (22.4)	97 (22.0)	
Severe (NIHSS >10)	419 (14.6)	197 (18.1)	129 (13.6)	44 (11.1)	49 (11.1)	
IV Thrombolysis given	396 (13.8)	151 (13.9)	122 (12.9)	59 (14.9)	64 (14.5)	0.74
Complications during admission	155 (5.4)	80 (7.4)	42 (4.4)	17 (4.3)	16 (3.6)	0.004
TOAST Classification						
Small Vessel Disease	1325 (46.2)	450 (41.4)	442 (46.7)	191 (48.1)	242 (55.0)	<0.001
Large Vessel Disease	561 (19.5)	207 (19.1)	193 (20.4)	83 (20.9)	78 (17.7)	
Cardioembolic	561 (19.5)	252 (23.2)	179 (18.9)	69 (17.4)	61 (13.9)	
Stroke of Determined Origin	250 (8.7)	108 (9.9)	82 (8.7)	25 (6.3)	35 (8.0)	
Stroke of Undetermined Origin	173 (6.0)	69 (6.4)	51 (5.4)	29 (7.3)	24 (5.5)	
Prognosis 90-Days (n= 2160)						
Good (mRS 0-2)	1642 (76.0)	586 (70.5)	554 (78.5)	240 (78.9)	262 (82.1)	<0.001
Poor (mRS 3-6)	518 (24.0)	245 (29.5)	152 (21.5)	64 (21.1)	57 (17.9)	
Mortality at 90-Days	56 (2.6)	26 (3.1)	18 (2.5)	5 (1.6)	7 (2.2)	0.52
One Year Outcome						
Recurrent Stroke (Ischemic or Hemorrhagic)	43 (2.0)	22 (2.7)	8 (1.1)	6 (2.0)	7 (2.1)	0.19
Myocardial Infarction (fatal or non-fatal)	8 (0.4)	2 (0.2)	3 (0.4)	2 (0.7)	1 (0.3)	0.76
All-Cause Mortality	67 (3.1)	32 (4.0)	22 (3.2)	6 (2.0)	7 (2.1)	0.24
Cardiac Arrest	11 (0.5)	5 (0.6)	4 (0.6)	0	2 (0.6)	0.62
Cardiac Revascularization (CABG or PCI)	9 (0.4)	2 (0.2)	3 (0.4)	3 (1.0)	1 (0.3)	0.36
Total MACE at one year	119 (4.1)	56 (5.2)	34 (3.6)	14 (3.5)	15 (3.4)	0.21

Triglyceride values – Low normal – 1.1 mmol/L or less, High normal- 1.2-1.7 mmol/L, Borderline High- 1.8-2.2 mmol/L, and High levels- 2.3 mmol/L or more. IV- intravenous, NIHSS- National Institute of Health Stroke Scale, RBS – Random blood sugar, HDL- High density lipoprotein, LDL Low density lipoprotein, BMI- Body Mass Index, mRS- Modified Rankin Score, CABG- Coronary artery bypass Graft, PCI- Percutaneous Coronary Intervention, MACE- Major Cardiac Adverse Event

Supplementary Table 3: Multivariate logistic regression analysis showing factors associated ischemic strokes of small vessel disease etiology .

Variable	Adjusted Odds Ratio	95.0% CI		P -value
		Lower	Upper	
**Age (Years)**	**1.02**	**1.01**	**1.02**	**<0.001**
**Gender**	**1.18**	**1.03**	**1.36**	**0.02**
**NIHSS on admission**	**0.88**	**0.87**	**0.89**	**<0.001**
**Hypertension**	**1.74**	**1.52**	**1.96**	**<0.001**
**Coronary Artery Disease**	**0.54**	**0.45**	**0.63**	**<0.001**
**Atrial Fibrillation**	**0.31**	**0.24**	**0.39**	**<0.001**
**Hypertriglyceridemia**	**1.09**	**1.05**	**1.15**	**<0.001**

Results were adjusted to diabetes, dyslipidemia, obesity and prior stoke variables.

Multivariate logistic regression analysis where hypertriglyceridemia was taken as continuous variable for small vessel disease demonstrated that hypertriglyceridemia was independently associated with small vessel disease after adjusting other important independent variables such as diabetes, dyslipidemia, obesity and prior stroke as well as age, sex, HTN, NIHSS on admission, coronary artery disease, and atrial fibrillation (Supplementary table).
